# Comparative Study of Powder Carriers Physical and Structural Properties

**DOI:** 10.3390/pharmaceutics14040818

**Published:** 2022-04-08

**Authors:** Klára Kostelanská, Barbora Blahová Prudilová, Sylva Holešová, Jakub Vlček, David Vetchý, Jan Gajdziok

**Affiliations:** 1 Department of Pharmaceutical Technology, Faculty of Pharmacy, Masaryk University, Palackého Třída 1946/1, 612 00 Brno, Czech Republic; 507255@muni.cz (K.K.); vetchyd@pharm.muni.cz (D.V.); 2 Department of Physical Chemistry, Faculty of Science, Palacký University Olomouc, Tř. 17. Listopadu 1192/12, 771 46 Olomouc, Czech Republic; barbora.blahovaprudilova@upol.cz (B.B.P.); jakub.vlcek@upol.cz (J.V.); 3 Nanotechnology Centre, CEET, VŠB-Technical University of Ostrava, 17. Listopadu 2172/15, 708 00 Ostrava, Czech Republic; sylva.holesova@vsb.cz

**Keywords:** powder carriers, adsorption, aluminometasilicates, liquisolid systems, solid dosage form, pharmaceutical technology

## Abstract

High specific surface area (SSA), porous structure, and suitable technological characteristics (flow, compressibility) predetermine powder carriers to be used in pharmaceutical technology, especially in the formulation of liquisolid systems (LSS) and solid self-emulsifying delivery systems (s-SEDDS). Besides widely used microcrystalline cellulose, other promising materials include magnesium aluminometasilicates, mesoporous silicates, and silica aerogels. Clay minerals with laminar or fibrous internal structures also provide suitable properties for liquid drug incorporation. This work aimed at a comparison of 14 carriers’ main properties. Cellulose derivatives, silica, silicates, and clay minerals were evaluated for flow properties, shear cell experiments, SSA, hygroscopicity, pH, particle size, and SEM. The most promising materials were magnesium aluminometasilicates, specifically Neusilin^®^ US2, due to its proper flow, large SSA, etc. Innovative materials such as FujiSil^®^ or Syloid^®^ XDP 3050 were for their properties evaluated as suitable. The obtained data can help choose a suitable carrier for formulations where the liquid phase is incorporated into the solid dosage form. All measurements were conducted by the same methodology and under the same conditions, allowing a seamless comparison of property evaluation between carriers, for which available company or scientific sources do not qualify due to different measurements, conditions, instrumentation, etc.

## 1. Introduction

Research in pharmaceutical technology has focused on developing and using powder carriers as structural materials for innovative drug formulations [1]. Carriers usually show a homogenous structure, high specific surface area (SSA), suitable pore size for drug incorporation, and advantageous technological properties (flow, compression, etc.) [2,3,4]. Several promising materials have been investigated in recent years, but most have been dismissed due to their non-biocompatibility or limited final processability [5]. Materials used as carriers for preparing dosage forms include microcrystalline cellulose (MCC); magnesium aluminometasilicates; clay minerals; colloidal silicon dioxide; and some others [5]. The porous structure of these materials allows the adsorption of the drug and its subsequent release in a predictable manner. Due to the ability to adsorb a drug, these materials have been used in the preparation of liquisolid systems (LSS) or solid self-emulsifying delivery systems (s-SEDDS) [2]. After incorporating the liquid component into the material’s structure, a solid system with suitable properties (flow, compressibility, etc.) for subsequent processing into a solid dosage form is formed. The penetration of the liquid into the pores is influenced by several properties of both the liquid (bulk and molecular) and the carrier (geometric and surface). Upon contact of the dosage form with the solvent (dissolution medium), the active substance contained in the pores or on the surface is washed out or dissolved. Subsequently, the active substance diffuses through the pores filled with the dissolution medium [5,6].

LSS represent modern formulations in which the drug in a liquid form is incorporated into the porous structure of the carrier. The resulting free-flowing and compressible solid system shows suitable properties for the next technological processing into the final dosage form (capsules, tablets, pellets, etc.) [2,5]. The penetration of the liquid and its subsequent adsorption onto the structure of the carrier material depends on the volume and physico-chemical properties of the liquid and the structural and surface properties of the carrier. When the LSS comes into contact with the dissolution medium, the already dissolved active pharmaceutical ingredient (API) is washed out from the carrier surface and released. Subsequently, the drug diffuses through the internal pores of the carrier filled with a dissolution medium [2,7,8].

The liquisolid technique for enhancing drug bioavailability has been used in many studies [9,10,11,12]. In the study by Chella et al. [13], there was the aim of enhancing the dissolution profile of valsartan (antihypertensive). Microcrystalline cellulose (Avicel^®^ PH 102) was used as the carrier, propylene glycol as the solvent, and Aerosil^®^ 200 as the coating material. After 30 min of the dissolution study, twice more valsartan was released from LSS than conventional tablets [13]. Komala et al. [14] prepared LSS with raloxifene hydrochloride (a selective estrogen receptor modulator) to improve solubility and permeation in the gastrointestinal tract. The dissolved drug was used in different concentrations (20 and 30% *w*/*w*) and adsorbed onto the carrier (Avicel^®^ PH 102). Aerosil^®^ 200 (colloidal silicon dioxide) was used as the coating material. Ex vivo tests on rat intestinal tissues showed improved drug permeation due to the ability of non-volatile solvents to increase intestinal permeability [14]. The work of Hentzschel et al. [15] aimed to substitute Avicel^®^ as a porous carrier by an excipient with high SSA and appropriate flow properties for LSS formulation. Carriers such as Avicel^®^ PH 102, Fujicalin^®^ (dicalcium phosphate), and Neusilin^®^ US2 (agglomerated magnesium aluminosilicate) were tested. Tocopherol acetate (vitamin) was used as the model drug. In this study, it was proven that the mentioned carriers have different adsorption capacities. The use of a highly sorptive excipient allows for the preparation of LSS containing higher doses of poorly soluble drugs, wherein large amounts of a liquid vehicle are usually needed for its dissolving. Using Neusilin^®^ as a carrier, the tocopherol acetate adsorption capacity was increased by 47% [15]. In the study by Sheth and Jarowski [16], it was proven that Syloid^®^ 244FP could be used as the carrier and the coating material for the formulation of LSS containing polythiazide (diuretic) [16]. LSS with montmorillonite carrier (clay mineral) was prepared by intercalation of ibuprofen (NSAID) into the clay’s structure. Dissolution tests showed that montmorillonite could be used as a carrier for sustained release of ibuprofen after oral administration [17].

Other formulations that can potentially enhance the bioavailability of poorly soluble drugs represent s-SEDDS. For the preparation of s-SEDDS, an isotropic mixture of oils and nonionic emulsifiers are usually used. Subsequently, the SEDDS is adsorbed onto the powder carrier [18,19]. A prepared dosage form can release the lipophilic drug. It self-emulsifies in the gastrointestinal tract due to the present fluid and its movements [18,19]. Yi et al. [20], in their research about s-SEDDS, adjusted the release of nimodipine (a selective calcium channel blocker) using a porous carrier [20,21]. Kamel et al. [22] prepared SEDDS with rutin (flavonoid). As excipients, emulsifier, co-emulsifier, and oil were used. The formed emulsion system was adsorbed onto powder carriers: Neusilin^®^ US2, Fujicalin^®^, and F-melt^®^. During the dissolution study, 90% of the drug was released within the first 15 min [22]. Aerosil^®^ 200 as a carrier was used in the study of Bhagwat et al. [23]. They prepared s-SEDDS with telmisartan (angiotensin II antagonist). It was proven that the formulated powder blend had sufficient flowability for next processing and that s-SEDDS can serve as formulations with increased dissolution rate and higher drug bioavailability [23]. Gumaste et al. [24] compared the suitability for s-SEDDS preparation of six silicates. Satisfactory results were obtained only when using Neusilin^®^ US2 due to its acceptable compressibility [24].

As shown in the above-mentioned research, a suitable powder carrier plays a vital role in formulating modern solid dosage forms with incorporated drugs in the liquid state. The topic of this work is a comparison of the main properties of 14 powder carriers: cellulose derivatives (Avicel^®^ PH 101, Methocel^®^ E4M, Methocel^®^ K100 LV), silicas and silicates (Aerosil^®^ 200, FujiSil^®^, Neusilin^®^ NS2N, Neusilin^®^ S2, Neusilin^®^ US2, Neusilin^®^ UFL2, Sipernat^®^ 22S, Syloid^®^ 244 FP, Syloid^®^ XDP 3050), and clay minerals (Bentonite, Vermiculite). These materials were evaluated under the same conditions for flow properties, including angle of slide, bulk and tap density, flow rate through the orifice, shear cell experiments, specific surface area, moisture content, hygroscopicity, pH leaching, particle size (measured by laser diffraction), true (pycnometric) density, porosity, and SEM structure. There is no similar comparative technical study that summarizes data regarding carriers’ properties. This fact negatively influences their correct selection for the intended use. The novelty of this study is in the evaluation of carrier materials by the same methodology and under the same conditions. Obtained data may help choose a suitable powder carrier before formulating a specific dosage form such as LSS or s-SEDDS.

## 2. Materials and Methods

### 2.1. Materials

For this work, powder materials from the group of (I) Cellulose derivatives: Avicel^®^ PH 101 (FMC Bio-Polymer, Cork, Ireland); Methocel^®^ E4M and Methocel^®^ K100LV (Colorcon Ltd., Dartford, UK); (II) Silicas and Silicates: Aerosil^®^ 200 (Evonik Industries AG, Essen, Germany); FujiSil^®^ (Fuji Chemical Industries Co., Ltd., Tokyo, Japan); Neusilin^®^ NS2N, Neusilin^®^ S2, Neusilin^®^ US2, Neusilin^®^ UFL2 (all Fuji Chemical Industries Co., Ltd., Tokyo, Japan); Sipernat^®^ 22S (Evonik Industries AG, Essen, Germany); Syloid^®^ 244FP and Syloid^®^ XDP 3050 (Grace Materials Technologies, Davenport, IA, USA); and (III) clay minerals: Bentonite (deposit, Ivančice, Czech Republic) and Vermiculite (deposit Santa Luzia, Brasil)—both milled in a planetary mill (Fritsch Pulverisette 7, Fritsch, Idar-Oberstein, Germany)—were selected.

### 2.2. Methods

#### 2.2.1. Particle Size

Particle size was evaluated on the basis of the volume principle by laser diffraction (LA-960, Horiba, Japan) using denatured alcohol as a liquid medium. Measurements were carried out three times (each sample was repeatedly prepared and measured; values are expressed as means). The most important value was the mean particle size. Other parameters were median size (D_50_), D_10_, and D_90_ (diameters of samples at the 50th, 10th, and 90th percentiles of the cumulative percent undersize plot, respectively). Another calculated parameter showing the width of the size distribution was span. The span of volume-based size distribution is defined as [25]:

(1) $$\mathrm{span}=\;\left(D_{90}-D_{10}\right)/D_{50}$$

#### 2.2.2. Scanning Electron Microscope (SEM)

The surface structure and particle morphology of the porous materials were determined by SEM. The samples were placed on aluminum stubs with double-side adhesive carbon tape, coated with a 10 nm gold layer using sputtering equipment (Quorum Technologies, Lews, UK), and observed using a scanning electron microscope (MIRA3, Tescan Brno, s.r.o., Brno, Czech Republic). Obtained signals of the samples were produced by secondary electrons (SE) at 5 kV voltage and 500× magnification.

#### 2.2.3. Specific Surface Area

The nitrogen adsorption–desorption isotherm of the samples was tested using a surface area and pore size analyzer (Thermo Scientific Surfer, Milan, Italy) to obtain information on the specific surface area (SSA), pore size distribution (W_BJH_, W_HK_), and total pore volume (V_t_). The silica and silicate samples were outgassed at 150 °C for 48 h under vacuum; other cellulose and clay mineral samples were outgassed at 70 °C for 12 h under vacuum. The specific surface area was calculated according to the Brunauer–Emmett–Teller (BET) method. The pore size distribution of mesopores was obtained from the corresponding adsorption branch at a relative pressure of P/P0 = 0.3–0.95 by using the Barrett–Joyner–Halenda (BJH) approach. The pore size distribution of micropores was obtained from the corresponding adsorption branch at a relative pressure of P/P0 = 0–0.35 using the Horvath and Kawazoe (HK) approach. Moreover, the total pore volume (V_t_) was evaluated from N_2_ adsorption at the relative pressure of 0.92 [26].

#### 2.2.4. True Density and Porosity

The powder materials’ true density was evaluated by the gas displacement technique using the helium pycnometer (Pycnomatic ATC, Ing. Prager Elektronik Handels GmbH, Wolkersdorf im Weinviertel, Austria), according to Ph. Eur. Porosity was calculated according to Equation (2) [27].

(2) $$\mathrm{porosity}=\;\left(1-\rho_{\mathrm{bulk}}/\rho_{\mathrm{true}}\right)\cdot 100$$

#### 2.2.5. Moisture Content

The percentage of moisture content in the powder materials was evaluated by a halogen moisture analyzer (Mettler Toledo, HX204, Greifensee, Switzerland) under the given conditions: standard drying program, drying temperature 105 °C, switch-off criterion 1 mg·50 s^−1^. Measurements were carried out three times. Results are reported as start points (time 0—measured immediately after opening the original packaging) with hygroscopicity data.

#### 2.2.6. Hygroscopicity

The hygroscopicity was assayed in a constant climate chamber (Binder, KBF 240, Tuttlingen, Germany) under given conditions: temperature 40 °C, relative humidity 75%, duration 30 days. Three grams of samples in glass vials were placed into a climate chamber and tested after 0.25, 0.5, 1, 3, 8, 24, 72, 120, 168, and 720 h by a halogen moisture analyzer (Mettler Toledo, HX204, Greifensee, Switzerland). Measurements were carried out three times.

#### 2.2.7. pH Leaching

pH leaching was evaluated as a pH of 2% water dispersion of the tested carrier. The distilled water needed for measurement was degassed by 5 min boiling and subsequent 5 min sonification. The dispersion pH was tested (after 5 min standing) using a surface pH microelectrode connected to a pH meter (pH 210, Hanna Instruments, Curepipe, Mauritius). Measurements were carried out three times.

#### 2.2.8. Flow Properties

The flow rate through the orifice of powder materials was measured by a flowability tester (Ing. Havelka, Brno, Czech Republic) according to Ph. Eur. [28], with a 25 mm orifice diameter. Measurements were carried out three times, and the results are presented as mean values ± standard deviations.

Bulk and tapped volumes were tested in a tapped density tester (SVM 102, Erweka GmbH, Langen (Hessen), Germany) and served to evaluate bulk and tapped densities, Hausner ratio (HR), and compressibility indexes (CI) according to Ph. Eur. [28].

#### 2.2.9. Angle of Slide

The angle of the slide was evaluated with a powder sample (10 g) placed on one end of a metal (stainless steel) plate with a chrome-plated surface. This end was gradually raised until the plate on the horizontal surface formed an angle at which the sample was about to slide [29]. Measurements were carried out three times, and the results are presented as mean values ± standard deviation.

#### 2.2.10. Shear Cell Experiment

The powder rheology of each sample was measured by an FT4 Powder rheometer (Freeman Technology, Tewkesbury, UK). All samples were loaded into a 25 mL shear cell. Measurements were carried out under the laboratory temperature of 23 °C, atmospheric pressure, and relative humidity of 43%. The Mohr’s circles and yield locus of the studied powder materials as obtained by shear cell experiments using 9 kPa consolidation stress allowed for the description of flow properties such as cohesion, flow function coefficient (FFc), angle of internal friction (AIF), and relative flow index (Relf). Jenike proposed FFc to describe the powder’s ability to flow, which is characterized by the ratio of the consolidation stress σ_1_ (the major principal stress MPS, received from Mohr stress circle of the steady-state flow for applied normal consolidation stress) to the unconfined yield strength σ_c_ (the maximum normal stress value which a solid has, also UYS) [30]. FFc can be calculated using Equation (3) [31].

(3) $$\mathrm{FFc}=\sigma_{1}/\sigma_{c}=\mathrm{MPS}/\mathrm{UYS}$$

An angle of internal friction determines the powder’s flowability (easily or poorly flowing) and ranges from 0° to 90° [32]. The relative flow index Relf proposed by Peschl classifies the powder’s cohesion level. The Relf was calculated using Equation (4) [33].

(4) $${\mathrm{Rel}}_{}f=(\sigma_{1}-\sigma_{2})/\sigma_{c}=\;\left(\mathrm{MPS}-\mathrm{MCS}\right)/\mathrm{UYS}$$

where σ_2_ is the minor principal consolidation stress at a steady flow. Measurements were carried out three times.

## 3. Results and Discussion

This work aimed to compare the physical and structural properties of 14 powder carriers potentially suitable for use in pharmaceutical technology. Powder materials were tested for flow properties, true density and porosity, particle size characterized by laser diffraction, specific surface area, moisture content, hygroscopicity, pH leaching, shear cell experiments, and scanning electron microscopy.

### 3.1. Particle Size

**Figure 1:**
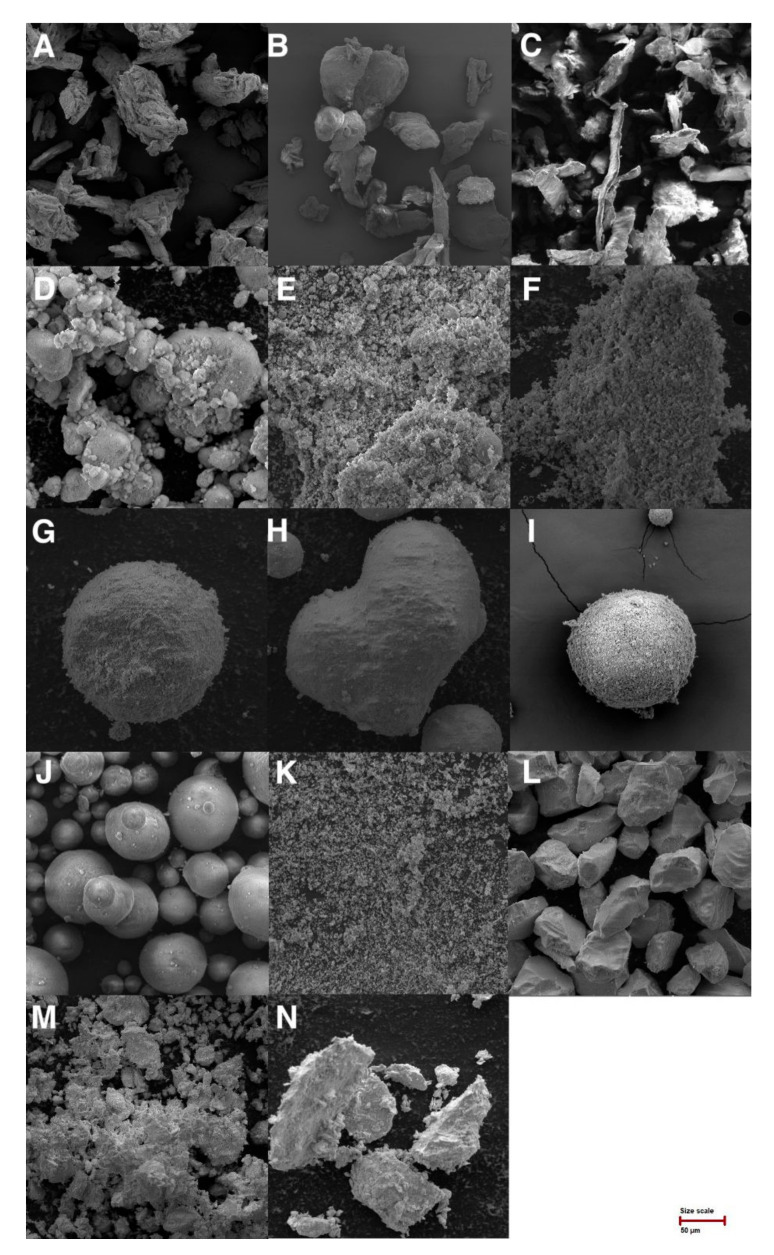
SEM images of Avicel^®^ PH 101 (**A**), Methocel^®^ E4M (**B**), Methocel^®^ K100LV (**C**), Aerosil^®^ 200 (**D**), Sipernat^®^ 22S (**E**), Neusilin^®^ UFL2 (**F**), Neusilin^®^ NS2N (**G**), Neusilin^®^ S2 (**H**), Neusilin^®^ US2 (**I**), FujiSil^®^ (**J**), Syloid^®^ 244FP (**K**), Syloid^®^ XDP 3050 (**L**), Bentonite (**M**), and Vermiculite (**N**) at magnification 500×.

The size of the particles, or their distribution, impacts the technological processability and content uniformity of the final solid dosage forms. Components in solid dosage forms tend to homogenize better when they are of comparable particle size [34]. On the other hand, smaller particles can benefit from adhering to the surface of predominantly presented larger particles and coating them [35]. In general, materials with large particles show better flowing properties. Carriers with small particle sizes usually offer higher sorptive capacity due to large surfaces, but their flow and processability are limited. Mean particle size (MPS), span, D_10_, D_50_ (median particle size), and D_90_ were analyzed by laser diffraction (Table 1). The measured values could be influenced by the shape of the particles (laser diffraction interpolates the signals to a spherical shape), so the particle size distribution values should be confirmed, e.g., by image analysis from a scanning electron microscope (Figure 1) [36].

Cellulose derivatives showed particle size data confirming the manufacturers’ specifications. For Avicel^®^ PH 101, the manufacturer indicated the median particle size about 50 µm, which was confirmed (52.5 µm) (Table 1) [37]. The particle size of the two types of Methocel^®^ was not similar, being visible on SEM images (Figure 1). Methocel^®^ E4M (142.8 µm) contains more long fibrous particles than Methocel^®^ K100LV (74.3 µm) [38].

Furthermore, laser diffraction measurements were performed in silicas and silicates. The Aerosil^®^ (44.3 µm) is usually measured by laser diffraction as tightly coupled aggregates of Aerosil as very fine primary particles (nm size) [39,40]. Fujisil^®^ showed a mean particle size of 76.5 µm, whereas the manufacturer indicated 80 µm [41], and Syloid^®^ XDP was 3050 60.7 µm (manufacturers data 50 µm) [42]. The last two samples of silica, Sipernat^®^ 22S (13.3 µm) and Syloid^®^ 244 FP (2.4 µm), showed very fine particles (Table 1) that were connected to their worst flow properties (Table 1). Both values of these materials followed that of the manufacturer (Sipernat^®^ 22S 14 µm; Syloid^®^ 244FP 3.5 µm) [42,43]. Together with Aerosil^®^, Neusilin^®^ UFL2 and Bentonite could be preferably used as coating materials due to their small particles capable of depositing on the surface of large carrier particles.

The largest particles of silicates were in Neusilin^®^ S2 (117.5 µm) and Neusilin^®^ US2 (108.4 µm). Another magnesium aluminometasilicate contained smaller particles, Neusilin^®^ NS2N (63.0 µm) and Neusilin^®^ UFL2 (3.5 µm). Measured values were confirmed by the manufacturer, who indicated for Neusilin^®^ S2 115 µm, Neusilin^®^ US2 106 µm, Neusilin^®^ NS2N 44–170 µm, and Neusilin^®^ UFL2 3.1 µm [44].

Clay minerals represented by Bentonite (11.8 µm) and Vermiculite (68.0 µm) corresponded to the SEM images.

Span represents an important parameter that expresses the width of the particle size distribution (the lower its value, the narrower the particle size distribution) [25]. The values of the measured materials ranged from 0.38 (Bentonite) to 2.14 (Neusilin^®^ NS2N) (Table 1). Samples of cellulose derivatives (Avicel^®^ PH 101 1.45, Methocel^®^ E4M 1.51), silicas, and silicates (Aerosil^®^ 200 1.54, Neusilin^®^ UFL2 1.26, Neusilin US2 1.42, Sipernat^®^ 22S 1.51, Syloid^®^ 244FP 0.82, Syloid^®^ XDP 3050 1.34), and clay minerals (Bentonite 0.38, Vermiculite 1.24) showed relatively low values of the span, indicating that the particles had uniform size [35]. Samples with higher values of the span as cellulose derivative (Methocel^®^ K100LV 1.75) and silicates (Fujisil^®^ 1.86, Neusilin^®^ NS2N 2.14, Neusilin^®^ S2 2.00) could cause problems during solid dosage form manufacturing due to their size non-uniformity.

### 3.2. Scanning Electron Microscopy (SEM)

SEM images of cellulose derivatives (A–C), silica and silicates (D–L), and clay minerals (M–N) are visible in Figure 1. In the case of cellulose derivatives, Avicel^®^ PH 101 was observed in particular as cellulose microcrystals are packed tightly in the fiber direction in a compact structure resembling bundles of wooden matchsticks placed side by side [45]. The manufacturer indicates that Methocel^®^ K100LV contains longer, more fibrous particles than Methocel^®^ E4M [38]. This statement was confirmed in Figure 1 and using a measurement by laser diffraction where we measured mean particle size for Methocel^®^ E4M (27.71 µm) and Methocel^®^ K100LV (82.90 µm).

Silicas and silicates, especially FujiSil^®^, Neusilin^®^ NS2N, and Neusilin^®^ S2, showed spherical well-agglomerated particles. All four types of Neusilin^®^ used in this study were significantly different in the images. Images confirmed that three types of magnesium aluminometasilicates have different kinds of particles. Three types (NS2N, S2, US2) are available on the market in granular form, and one type (UFL2) in powder form [46]. Other materials showed nearly nonspherical particles. A comparison of Syloids^®^ (Figure 1) showed a difference in their particles. Syloid^®^ XDP 3050 was similar in appearance and particle size to the granulated form of aluminometasilicates. Reuzel et al. [47] claimed that Aerosil^®^ 200 and Sipernat^®^ 22S had a spherical primary particle shape [47]. In Figure 1, it is shown that the claim can be confirmed under higher magnification.

Clay minerals have several types of morphology. Bentonite shows the typical surface appearance called clay largely composition [48]. This applies to the relatively homogenous soils where most particles are characterized by a varied anisotropy of shape [36].

### 3.3. Specific Surface Area (SSA)

Specific surface area is one of the most important factors for selecting a powder material as a suitable carrier [42]. SSA is related to the ability of the material to absorb the drug onto its surface and in “open” pores. The higher this value is, the higher is the material absorption capacity [5]. The BET method was used to measure tested powders’ SSA (Table 2). The highest measured SSA values were obtained for silicate samples, mainly for new porous silica material available on the market called FujiSil^®^ (SSA 374.55 ± 4.48 m^2^/g; the size of mesopores 9.33 nm, micropores 0.41 nm, and pore volume 0.46 cm^3^/g). This indicated its promising possible use as a porous carrier [49]. Some of the tested magnesium aluminometasilicates showed higher values than those that were presented by the manufacturers. The manufacturer indicated that Neusilin^®^ UFL2 (SSA 350.33 ± 2.88 m^2^/g; the size of mesopores 7.62 nm, micropores 0.45 nm) and Neusilin^®^ US2 (SSA 342.16 ± 2.72 m^2^/g; the size of mesopores 7.99 nm, micropores 0.44 nm) should have SSA 300 m^2^/g and Neusilin^®^ S2 (SSA 168.82 ± 1.04 m^2^/g; the size of mesopores 5.01 nm, micropores 0.46 nm) 110 m^2^/g [50]. The results of this method strongly depend on the conditions of sample handling, such as the time or temperature of degassing. The accuracy of the measurements was confirmed in the case of colloidal silicas Aerosil^®^ 200 (SSA 190.48 ± 1.74 m^2^/g; the size of mesopores 7.04 nm, micropores 0.50 nm, and pore volume 0.24 cm^3^/g) and Sipernat^®^ 22S (SSA 188.92 ± 2.06 m^2^/g; the size of mesopores is 9.70 nm, micropores 0.48 nm and pore volume is 0.24 cm^3^/g). The results were compared to the study of Reuzel et al. [47], where Aerosil^®^ 200 showed SSA 200 m^2^/g and Sipernat^®^ 22S 190 m^2^/g [47]. Regarding the pore size, it is important to note that Syloids are highly porous (Syloid^®^ 244FP, 358.73 ± 3.26 m^2^/g; Syloid^®^ XDP 3050 289.32 ± 2.29 m^2^/g) and have a pore size 2-50 nm, as confirmed by the measurements (Syloid^®^ 244FP mesopores 10.66 nm, micropores 0.50 nm; Syloid^®^ XDP 3050 mesopores 10.58 nm, micropores 0.50 nm) expressed in Table 2 [51]. Pore volume was evaluated for pores smaller than 100 nm in diameter and was determined from desorption data [52]. The pore volume of the measured data ranged between 0.02 and 0.73 cm^3^/g (Table 2). In the study of Westermarck et al. [52], two types of measurements of pore volume were compared, and we observed that granules had a higher value of pores than powders [52]. This condition was partially met because the agglomerated magnesium aluminometasilicates (Neusilin^®^ NS2N 0.67 cm^3^/g, Neusilin^®^ S2 0.30 cm^3^/g, Neusilin^®^ US2 0.69 cm^3^/g) had one of the highest pore volume values (Table 2).

The lowest measured value was Vermiculite (SSA 15.88 ± 0.30 m^2^/g; the size of mesopores 3.34 nm, micropores 0.38 nm, and pore volume 0.02 cm^3^/g).

This test was not applicable for cellulose derivatives. Relatively low SSA of cellulose derivatives could cause the inability to measure this value in this study. Some research groups that used the BET technique obtained several pieces of specific surface area data. It was found that the value of SSA of cellulose derivatives usually ranges between 1 and 20 m^2^/g [42].

### 3.4. True Density and Porosity

The density of the powder is mainly related to the properties such as dilution potential and the size of the final solid dosage form (compressing of denser powder leads to the possible reduction of unsuitable properties of API) [53]. Experimentally measured values of true density were in the range from 1.29 ± 0.00 g/cm^3^ to 2.66 ± 0.02 g/cm^3^ (Table 7). The lowest true density was observed for cellulose derivatives (Avicel^®^ PH 101—1.58 ± 0.00 g/cm^3^, Methocel^®^ E4M—1.29 ± 0.00 g/cm^3^, and Methocel^®^ K100LV—1.33 ± 0.00 g/cm^3^). The literature indicates true density for MCCs from 1.51 to 1.67 g/cm^3^, while the true density of a perfect cellulose crystal is between 1.58 g/cm^3^ and 1.60 g/cm^3^ [54]. The measured MCC sample confirms this (Table 3). The highest true density was observed for a sample of silica Aerosil^®^ 200 (2.66 ± 0.02 g/cm^3^) as expected for amorphous precipitated material [55]. Values of other silicas and silicates did not differ significantly from each other, and their values ranged from Neusilin^®^ NS2N (2.14 ± 0.02 g/cm^3^) to Syloid^®^ 244FP (2.44 ± 0.02 g/cm^3^). From Table 3, it is evident that the highest values reached silicas and silicates. The literature shows that highly porous materials usually have high true density (helium reaches very small pores with the open character) [56]. High density values also reached clay minerals Bentonite (2.42 ± 0.00 g/cm^3^) and Vermiculite (2.64 ± 0.00 g/cm^3^) (Table 3).

Porosity values greater than 90% indicate that the particle structure is very porous, usually correlated with a low bulk density. The highest porosity was measured by Aerosil^®^ 200 (98.87%) (Table 3). High porosity values are also typical for other silicates (Table 7) [51]. Powder porosity is influenced by the particles’ size, shape, and especially specific surface area [57]. Samples of cellulose derivatives showed porosity values of 65.11% (Methocel^®^ K100LV), 75.94% (Methocel^®^ E4M), and 77.85% (Avicel^®^ PH 101). The lowest porosity of all three tested groups of materials was observed in clay minerals (Bentonite 68.60%, Vermiculite 64.02%) (Table 3).

### 3.5. Moisture Content, Hygroscopicity, and pH Leaching

The moisture content of solid-state pharmaceutical products is one of the main factors that affect drug stability, mechanical properties, processability, etc. [58]. In the case of powder materials, the values of moisture content are connected to the porosity because water can fill the open pores of the material and decrease its porosity. Values of moisture content for all tested powder materials ranged between 1.6 and 8.2% (Table 4). Relatively low values reached cellulose derivatives (Avicel^®^ PH 101 2.9%). As for water-swellable cellulose derivatives, Methocel^®^ showed higher values (Methocel^®^ E4M 3.6%, Methocel^®^ K100LV 4.7%) compared to Avicel^®^ PH 101, which is related to their higher hygroscopicity [59]. Low moisture content values were also reported for some silicas (mainly Syloid^®^ 244FP 3.6% and Syloid^®^ XDP 3050 3.4%). Syloids can be used to increase the stability of moisture-sensitive APIs [60] and are recommended to improve the physical stability of the dosage form (moisture reduces the feasibility of the drug formulation, reduces the flowability of the pharmaceutical composition, and reduces tablet hardness) [60].

Hygroscopicity is an unfavorable property of many materials used in pharmaceutical technology. Hygroscopicity can reduce the adsorption capacity of the drug due to adsorbed water, change the physical properties of used materials (agglomeration), and sometimes lead to specific requirements of processing conditions and packaging to ensure stability of the drug. From Table 4, it is evident that the moisture content increased from time 0 to 720 h for all the tested materials stored in conditions of increased temperature (40 °C) and high relative humidity (75%).

Callahan et al. [61] showed that cellulose derivatives belong to a slightly hygroscopic materials. The moisture content did not increase at a relative humidity below 80%. After storage for one week above 80% RH, the increase in moisture content was less than 40% [61]. This was not completely confirmed in this study. An increase in the moisture content of Avicel^®^ PH 101 + 4.4%, Methocel^®^ E4M + 4.1%, and Methocel^®^ K100LV + 3.5% during 720 h of testing (Table 4) was found.

The highest hygroscopicity showed silicates, mainly Neusilin^®^ US2, by which the hygroscopicity increased by 10.3% (0 h 4.6%, 720 h 14.9%) (Table 4). Neusilin^®^ itself is a relatively hygroscopic material due to capillary condensation of water in its pores [52]. In the study of Callahan et al. [61], other silicates were classified, mainly magnesium aluminometasilicates, which showed that during storing at 75% RH, values of moisture content increased by about 15% (measured values in this study after 720 h: Neusilin^®^ NS2N 9.8%, Neusilin^®^ S2 11.2%, Neusilin^®^ UFL2 13.8%). According to the study, magnesium aluminometasilicates belong to the moderately hygroscopic materials group [52]. This study also tested Aerosil^®^ 200, classified as non-hygroscopic or slightly hygroscopic [61]. This was also confirmed in this study, where its moisture content was increased only by 1.4% (0 h 1.6%, 720 h 3.0%) (Table 4).

Clay minerals showed higher hygroscopicity values under the specified conditions (40 °C, 75% RH). Especially for Bentonite, the moisture content increased by 2.3% (0 h 7.2%, 720 h, 9.5%) (Table 4). Similar observations were found in the study presented by Chen et al. [62], where ambient air temperature and humidity impacted a sharp increase of moisture content of clay minerals [62].

Carrier’s pH can influence the drug stability, its transition between salt-base, its compatibility with other materials, etc. All tested powder materials showed neutral or slightly basic pH (Table 5). Experimentally measured pH of Avicel^®^ PH 101 was marginally higher (pH 7.3) than what was indicated by the manufacturer (pH 5.0–7.0) [58].

Silicates, mainly magnesium aluminometasilicates, contain -OH groups associated with Si, Mg, and Al in their structure, leading to different acidic and basic strengths [63]. The manufacturer indicated that Neusilin^®^ US2 (pH 6.9) and Neusilin^®^ UFL2 (pH 6.9) have a neutral pH, and Neusilin^®^ S2 (pH 9.4) and Neusilin^®^ NS2N (pH 8.3) have an alkaline pH, which was proven [46]. The same findings were observed for other silicas and silicates, such as Aerosil^®^ 200 (pH 6.3), where producer declared that the pH range should be 0–7.5 [39]. The study of Reuzel et al. [47] evaluated the physical properties of some powder materials such as silicate Sipernat^®^ 22S. It was stated that this material has a pH 6.3, which is slightly lower than that measured in this study (pH 7.4) (Table 5) [47]. One of the most alkaline pH was observed, focusing on clay minerals, especially Bentonite (pH 9.5). This was also reported in the study by Kaufhold et al. [64], which showed that the pH of this clay is in the range of 8.5–10.0 [64].

### 3.6. Flow Properties

Flow properties of powder materials were assessed by methods based on the material mobility, e.g., the ability of particles to migrate, such as flow through the orifice (flowability), angle of slide, and parameters such as CI and HR [28].

Poor flow is influenced by many factors (surface texture, particle size, internal friction, density, moisture content, etc.) [65], leading to failure in the next material processability or pharmacopoeial requirements for mass and content uniformity of the final dosage form. In general, larger particles with a smooth surface, regular shape, and higher density show better flowability [66]. Appropriate flowability was observed for microcrystalline cellulose Avicel^®^ PH 101 (3.4 ± 0.4 s) and Methocel^®^ E4M (3.2 ± 0.4 s). Another type of hydroxypropyl methylcellulose, Methocel^®^ K100LV, showed a higher flowability value (9.0 ± 0.6 s), although Methocel^®^ E4M has much smaller particles (27.7 µm) than Methocel^®^ K100LV (82.9 µm) (Table 1). This could be caused by the higher density (Table 6) and lower porosity (Table 3) of Methocel^®^ E4M and their differences in molecular weight and methoxy vs. hydroxypropoxy content [46]. The best flowability of all tested samples was manifested by FujiSil^®^ (1.5 ± 0.1 s) (Table 6), which is by the manufacturer described as a free-flowing powder [41]. From Table 5, it is evident that Neusilin^®^ UFL2 (not measurable) had worse flowability due to its fine powder form (Table 6) in comparison with agglomerated forms of other aluminometasilicates: Neusilin^®^ US2 (11.8 ± 1.0 s), Neusilin^®^ S2 (4.4 ± 0.2 s), and Neusilin^®^ NS2N (11.5 ± 0.2 s). Flowability testing was not applicable for materials: Aerosil^®^ 200, Bentonite, Neusilin^®^ UFL2, Sipernat^®^ 22S, and Syloid^®^ 244FP (Table 6), and their flow could be marked as interminable.

Bulk densities of all tested samples ranged between 0.03 g/cm^3^ (Aerosil^®^ 200) and 0.76 g/cm^3^ (Bentonite) (Table 6). In general, they correspond to the physical structure of the tested materials. Measured tapped densities were between 0.04 g/cm^3^ (Aerosil^®^ 200) and 1.03 g/cm^3^ (Bentonite). Indexes HR and CI (Table 5) are based on the ability of the powder to decrease its apparent density and are evaluated by the comparison of bulk and tapped density [28]. The measured values ranged from 1.15 (Neusilin^®^ S2) to 1.48 (Methocel^®^ K100LV) for HR and from 12.8% (Neusilin^®^ S2) to 32.3% (Methocel^®^ K100LV) for CI, which corresponds with pharmacopoeial characterization from “good” to “very poor” flow [28]. The best flow properties of the measured materials exhibited samples Neusilin^®^ S2 (HR 1.15; CI 12.8%) and Neusilin^®^ US2 (HR 1.19; CI 15.8%), which belong to agglomerated types of aluminometasilicates.

From the measured data of powder flow properties (Table 6), it is evident that most of the tested materials do not show good flow properties when used alone. Most of them are functional excipients to prepare solid dosage forms after their suitable combination with other excipients such as lubricants or incorporating a liquid phase into their structures [59,67]. For example, colloidal silica is the preferred coating material in the preparation of LSS because of its ability to adsorb the excess liquid from the carrier and ensure the good flowability of the created mixture [68]. Microcrystalline cellulose is commonly used as a carrier in LSS because of its promising sorptive properties, long-term utilization, low price, good stability, and availability in different particle sizes and moisture grades [59]. Magnesium aluminometasilicates are used in the pharmaceutical industry as carriers for solid dispersions to improve drug dissolution or to granulate oily formulations and increase formulation stability [68].

### 3.7. Angle of Slide

According to the study by Spireas et al., there is the appropriate value of the angle of slide 33° (usually evaluated for powders with already adsorbed liquid phase) [69]. This value was closest to Neusilin^®^ S2 (36.3 ± 1.2°) and FujiSil^®^ (37.3 ± 0.6°) (Table 7). Other powders showed higher values (39.3 ± 2.5°—53.3 ± 0.6°) of the angle of slide than the optimum, which indicated their worse flow properties [44]. The highest angle of slide showed Aerosil^®^ 200 (53.3 ± 0.6°). It is caused by its fluffy structure and fine aggregates (8.9 µm) [70]. A higher angle of slide was also reported by Neusilin^®^ UFL2 (43.3 ± 2.5°—lower than that presented by the manufacturer at 45.0°). It is caused by a powder structure and small particle size (measured 6.2 μm (Table 1); declared by the manufacturer at 3.1 μm) [46]. With decreasing particle size, the flow function line progressively worsened the flow properties [66].

### 3.8. Shear Cell Experiments

Shear properties inform how easily the consolidated powder starts to flow. The flow begins when the yield point of the powder is overcome. The yield point is affected by physical properties such as the size and shape of the particles, the moisture content in the material, or the number of flow additives [26]. All the measured powders were exposed to consolidation stress during handling, transport, and storage. This exposition can change mechanical interparticulate forces and density of the powder and impact the measurement [26,71].

The flow properties of tested powders were measured using a shear cell (Table 8). Cellulose derivatives Avicel^®^ PH 101 (FFc = 20), Methocel^®^ E4M (FFc = 24), and Methocel^®^ K100LV (FFc = 16) exhibited high values of flow function, indicating free-flowing character. These observed free-flowing behavior, especially in the case of Methocel^®^ E4M, was supported by the low value of angle of internal friction (36.2°) and low value of cohesion (0.193) [48]. However, Avicel^®^ PH 101 (36.7°) and Methocel^®^ K100LV (45.0°) indicated slightly cohesive behavior. This was confirmed by flowability testing (Table 6), where Methocel^®^ E4M was evaluated with its properties (3.21 ± 0.38 s) as the best in the group of cellulose derivatives. These results agree with the manufacturer, who declared better flow properties of Methocel^®^ E4M [49]. Results of the relative flow index proposed that Avicel^®^ PH 101 (Relf = 15) and Methocel^®^ K100LV (Relf = 18) were slightly cohesive, and Methocel^®^ E4M (Relf = 13) was a non-cohesive material.

Most tested silica and silicate powders were characterized as free or easy-flowing materials (Table 8). FujiSil^®^, Neusilin^®^ US2, Neusilin^®^ S2, and Syloid^®^ XDP 3050 were immeasurable on shear cell; even the higher consolidation pressure was used during the measurement (up to 15 kPa). This indicated the free-flowing character of the materials. As an easily flowing were evaluated silicate and silica Neusilin^®^ UFL2 and Sipernat^®^ 22S (FFc was 6 in both cases). These results did not correlate with flowability measurements (Table 6), where these two materials were immeasurable. The method with the standardized funnel is limited by the stagnation of the outflow when cohesive powders are tested [72]. Neusilin^®^ UFL2 and Sipernat^®^ 22S were evaluated as cohesive, according to the shear cell experiments. The case of the angle of internal friction showed the lowest values Aerosil^®^ 200 (27.9°) and Neusilin^®^ NS2N (19.2°). This was also related to the relative flow index, which indicated that incoherent samples have better flowability [62]. These claims also correlated with results of FFc for other silica and silicates such as Neusilin^®^ NS2N (FFc = 58), Syloid^®^ 244FP (FFc = 38), and Aerosil^®^ 200 (FFc = 16), which indicated that these were free-flowing materials.

Clay minerals were evaluated as rather cohesive with their relative FFc for Bentonite (FFc = 4) and Vermiculite (FFc = 5). The claim about the cohesive property was confirmed by the results of Bentonite flowability (Table 6), where this powder clogged the orifice. Clay minerals were evaluated as very cohesive with values of Bentonite (Relf = 3) and Vermiculite (Relf = 4). The cohesive behavior of the clay minerals powder materials was confirmed by several studies, for example, Broms et al. [48].

### 3.9. Graphical Visualization of Results

**Figure 2:**
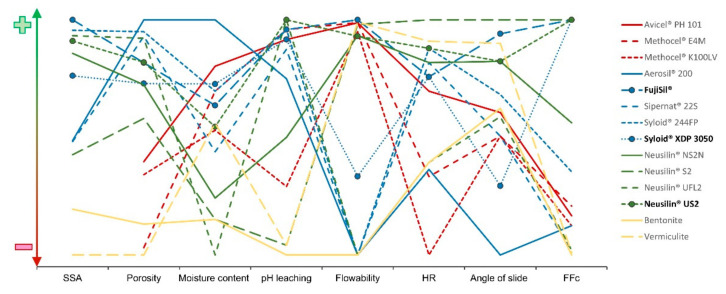
Comparison of selected results of powder carriers represented by graphical visualization.

Graphical visualization (Figure 2) was created from selected results to compare the tested carriers better. The *X*-axis represents tested parameters. The *Y*-axis was divided into ideal range (green color; sign +), acceptable range (orange color; the middle part of the graph), and non-acceptable range (red color; sign −). Each carrier is represented by one line.

## 4. Conclusions

Powder carriers represent materials useful for many applications in the pharmaceutical industry (formulations of LSS, s-SEDDS, etc.). The lack of comparative summarized data about their properties can limit their correct selection for the intended use. Fourteen available carrier materials were evaluated in this study. The materials with the most promising and balanced properties were evaluated as being magnesium aluminometasilicates (Neusilin^®^ US2). New materials on the pharmaceutical market, such as FujiSil^®^ or Syloid^®^ XDP 3050, were evaluated as promising porous carriers. Some of the powder materials with small particles and worse flow properties (Neusilin^®^ UFL2, Bentonite, and Aerosil^®^) could be advantageous as coating materials that cover the surface of primary carriers during the formulation of LSS or s-SEDDS.

## Figures and Tables

**Table 1:** Particle size of powder materials.

	MPS ^a^ (µm)	D_10_ (µm)	D_50_^b^ (µm)	D_90_ (µm)	Span
CELLULOSES					
Avicel^®^ PH 101	57.4	21.0	52.5	97.2	1.45
Methocel^®^ E4M	153.8	54.6	142.8	269.6	1.51
Methocel^®^ K100LV	89.3	36.4	74.3	166.7	1.75
SILICAS and SILICATES					
Aerosil^®^ 200	53.4	23.9	44.3	92.2	1.54
FujiSil^®^	86.4	20.6	76.5	125.8	1.86
Neusilin^®^ NS2N	71.8	11.1	63.0	145.8	2.14
Neusilin^®^ S2	170.6	46.8	117.5	281.8	2.00
Neusilin^®^ UFL2	6.2	2.1	3.5	6.5	1.26
Neusilin^®^ US2	110.8	33.2	108.4	187.5	1.42
Sipernat^®^ 22S	19.7	7.6	13.3	27.7	1.51
Syloid^®^ 244FP	2.5	1.5	2.4	3.5	0.82
Syloid^®^ XDP 3050	59.4	12.3	60.7	93.7	1.34
CLAY MINERALS					
Bentonite	11.9	9.8	11.8	14.2	0.38
Vermiculite	66.0	15.1	68.0	99.6	1.24

^a^ Mean particle size; ^b^ median particle size.

**Table 2:** Specific surface area, and values for mesopores, micropores, and pore volume.

	SSA ^a^ (m^2^/g)	Mesopores Radius (nm)	Micropore Radius (nm)	Pore Volume/ (cm^3^/g)
CELLULOSES				
Avicel^®^ PH 101	NA ^b^	NA ^b^	NA ^b^	NA ^b^
Methocel^®^ E4M	NA ^b^	NA ^b^	NA ^b^	NA ^b^
Methocel^®^ K100LV	NA ^b^	NA ^b^	NA ^b^	NA ^b^
SILICAS and SILICATES				
Aerosil^®^ 200	190.48 ± 1.74	7.04	0.50	0.24
FujiSil^®^	374.55 ± 4.48	9.33	0.41	0.46
Neusilin^®^ NS2N	323.56 ± 2.14	5.90	0.46	0.67
Neusilin^®^ S2	168.82 ± 1.04	5.01	0.46	0.30
Neusilin^®^ UFL2	350.33 ± 2.88	7.62	0.45	0.73
Neusilin^®^ US2	342.16 ± 2.72	7.99	0.44	0.69
Sipernat^®^ 22S	188.92 ± 2.06	9.70	0.48	0.24
Syloid^®^ 244FP	358.73 ± 3.26	10.66	0.50	0.63
Syloid^®^ XDP 3050	289.32 ± 2.29	10.58	0.50	0.58
CLAY MINERALS				
Bentonite	85.72 ± 1.37	2.23	0.39	0.07
Vermiculite	15.88 ± 0.30	3.34	0.38	0.02

^a^ Specific surface area; ^b^ not applicable.

**Table 3:** True density and porosity of powder materials.

	DT ^a^ (g/cm^3^)	Porosity (%)
CELLULOSES		
Avicel^®^ PH 101	1.58 ± 0.00	77.85
Methocel^®^ E4M	1.29 ± 0.00	65.11
Methocel^®^ K100LV	1.33 ± 0.00	75.94
SILICAS and SILICATES		
Aerosil^®^ 200	2.66 ± 0.02	98.87
FujiSil^®^	2.27 ± 0.02	92.51
Neusilin^®^ NS2N	2.14 ± 0.02	89.25
Neusilin^®^ S2	2.16 ± 0.01	84.26
Neusilin^®^ UFL2	2.34 ± 0.01	96.15
Neusilin^®^ US2	2.29 ± 0.02	92.58
Sipernat^®^ 22S	2.25 ± 0.02	96.44
Syloid^®^ 244FP	2.44 ± 0.02	97.13
Syloid^®^ XDP 3050	2.27 ± 0.02	89.43
CLAY MINERALS		
Bentonite	2.42 ± 0.00	68.60
Vermiculite	2.64 ± 0.00	64.02

^a^ True (pycnometric) density.

**Table 4:** Hygroscopicity of powder materials at specific times.

	MC ^a^ (%)										
	0 h	0.25 h	0.5 h	1 h	3 h	8 h	24 h	72 h	120 h	168 h	720 h
CELLULOSES											
Avicel^®^ PH 101	2.9	4.6	4.5	5.1	5.6	5.7	5.7	5.7	5.8	6.2	7.3
Methocel^®^ E4M	3.6	3.6	3.7	3.7	3.7	3.8	4.0	4.8	4.9	5.4	7.7
Methocel^®^ K100LV	4.7	4.5	4.6	4.7	5.0	5.1	5.1	5.2	7.1	7.7	8.2
SILICAS and SILICATES											
Aerosil^®^ 200	1.6	1.6	1.7	1.7	1.7	1.9	1.9	2.4	2.4	2.7	3.0
FujiSil^®^	4.0	4.1	4.3	4.8	4.8	4.9	5.0	5.0	5.6	6.0	7.8
Neusilin^®^ NS2N	6.6	7.0	7.7	7.7	7.8	7.9	8.4	8.5	8.9	9.5	9.8
Neusilin^®^ S2	7.2	7.4	7.7	7.7	7.9	8.5	8.6	8.6	9.2	9.2	11.2
Neusilin^®^ UFL2	8.2	8.2	8.4	8.5	8.5	8.6	9.0	9.3	10.7	12.6	13.8
Neusilin^®^ US2	4.6	8.1	8.5	8.7	8.9	8.9	9.2	9.4	9.6	10.6	14.9
Sipernat^®^ 22S	5.3	5.5	5.6	5.6	5.7	5.9	6.0	6.5	5.6	7.2	7.5
Syloid^®^ 244FP	3.6	4.2	4.2	4.3	4.7	4.8	4.9	5.0	5.0	5.5	8.6
Syloid^®^ XDP 3050	3.4	4.1	4.4	4.4	4.4	4.5	4.8	5.2	5.8	6.0	6.2
CLAY MINERALS											
Bentonite	7.2	7.2	7.3	7.4	7.7	7.8	7.9	8.0	8.2	8.6	9.5
Vermiculite	4.5	4.5	4.6	4.8	4.9	4.9	4.9	5.3	5.4	5.4	4.9

^a^ Moisture content.

**Table 5:** pH leaching of powder materials.

	pH
CELLULOSES	
Avicel^®^ PH 101	7.3
Methocel^®^ E4M	7.2
Methocel^®^ K100LV	8.8
SILICAS and SILICATES	
Aerosil^®^ 200	6.3
FujiSil^®^	7.2
Neusilin^®^ NS2N	8.3
Neusilin^®^ S2	9.4
Neusilin^®^ UFL2	6.9
Neusilin^®^ US2	6.9
Sipernat^®^ 22S	7.4
Syloid^®^ 244FP	7.2
Syloid^®^ XDP 3050	7.3
CLAY MINERALS	
Bentonite	9.5
Vermiculite	9.4

**Table 6:** Flow properties of powder materials.

	Fw ^a^ (s)	DB ^b^ (g/cm^3^)	DT ^c^ (g/cm^3^)	HR ^d^	CI ^e^
CELLULOSES					
Avicel^®^ PH 101	3.4 ± 0.4	0.35	0.45	1.25	19.7
Methocel^®^ E4M	3.2 ± 0.4	0.45	0.62	1.37	27.1
Methocel^®^ K100LV	9.0 ± 0.6	0.32	0.49	1.48	32.3
SILICAS and SILICATES					
Aerosil^®^ 200	∞ ^f^	0.03	0.04	1.36	26.7
FujiSil^®^	1.5 ± 0.1	0.17	0.21	1.23	18.9
Neusilin^®^ NS2N	11.5 ± 0.2	0.23	0.29	1.21	17.1
Neusilin^®^ S2	4.4 ± 0.2	0.34	0.40	1.15	12.8
Neusilin^®^ UFL2	∞ ^f^	0.09	0.13	1.35	25.9
Neusilin^®^ US2	11.8 ± 1.0	0.17	0.20	1.19	15.6
Sipernat^®^ 22S	∞ ^f^	0.08	0.10	1.21	17.6
Syloid^®^ 244FP	∞ ^f^	0.07	0.09	1.19	15.9
Syloid^®^ XDP 3050	100.3 ± 2.5	0.24	0.30	1.23	18.6
CLAY MINERALS					
Bentonite	∞ ^f^	0.76	1.03	1.35	26.0
Vermiculite	2.9 ± 0.2	0.95	1.13	1.18	15.4

^a^ Flow through the orifice (flowability); ^b^ bulk density; ^c^ tapped density; ^d^ Hausner ratio; ^e^ compresibility index; ^f^ infinite flow.

**Table 7:** Angle of slide of powder materials.

	θ_s_^a^ (°)
CELLULOSES	
Avicel^®^ PH 101	43.0 ± 3.0
Methocel^®^ E4M	44.7 ± 1.5
Methocel^®^ K100LV	44.7 ± 0.6
SILICAS and SILICATES	
Aerosil^®^ 200	53.3 ± 0.6
FujiSil^®^	37.3 ± 0.6
Neusilin^®^ NS2N	39.3 ± 2.5
Neusilin^®^ S2	36.3 ± 1.2
Neusilin^®^ UFL2	43.3 ± 2.5
Neusilin^®^ US2	39.3 ± 1.5
Sipernat^®^ 22S	44.7 ± 0.6
Syloid^®^ 244FP	41.7 ± 1.2
Syloid^®^ XDP 3050	48.3 ± 1.5
CLAY MINERALS	
Bentonite	42.7 ± 0.6
Vermiculite	38.0 ± 1.7

^a^ Angle of slide.

**Table 8:** Shear cell experiments.

	Cohesion (kPa)	FFc ^a^	AIF ^b^ (°)	Relf ^c^
CELLULOSES				
Avicel^®^ PH 101	0.204	20	36.7	15
Methocel^®^ E4M	0.193	24	36.2	18
Methocel^®^ K100LV	0.324	16	45.0	13
SILICAS and SILICATES				
Aerosil^®^ 200	0.271	16	27.9	11
FujiSil^®^	NA ^d^	NA ^d^	NA ^d^	NA ^d^
Neusilin^®^ NS2N	0.078	58	19.2	29
Neusilin^®^ S2	NA ^d^	NA ^d^	NA ^d^	NA ^d^
Neusilin^®^ UFL2	0.681	6	32.6	5
Neusilin^®^ US2	NA ^d^	NA ^d^	NA ^d^	NA ^d^
Sipernat^®^ 22S	0.712	6	32.5	5
Syloid^®^ 244FP	0.115	38	37.1	28
Syloid^®^ XDP 3050	NA ^d^	NA ^d^	NA ^d^	NA ^d^
CLAY MINERALS				
Bentonite	1.030	4	30.4	3
Vermiculite	1.440	5	35.2	4

^a^ Flow function; ^b^ angle of internal friction; ^c^ relative flow index; ^d^ not applicable.

## Data Availability

Data are contained within the article.
